# Dengue fever and insecticide resistance in *Aedes* mosquitoes in Southeast Asia: a review

**DOI:** 10.1186/s13071-021-04785-4

**Published:** 2021-06-10

**Authors:** Soon Jian Gan, Yong Qi Leong, Muhammad Fakrul Hakim bin Barhanuddin, Siew Tung Wong, Shew Fung Wong, Joon Wah Mak, Rohani Binti Ahmad

**Affiliations:** 1 International Medical University, 57000 Kuala Lumpur, Malaysia; 2 Monash University Malaysia, 47500 Subang Jaya, Selangor Malaysia; 3 Institute for Research, Development and Innovation (IRDI), International Medical University, 57000 Kuala Lumpur, Malaysia; 4 Institute for Medical Research, Jalan Pahang, 50588 Kuala Lumpur, Malaysia

**Keywords:** Dengue, *Aedes* mosquitoes, Prevalence, Insecticide resistance, Southeast Asia

## Abstract

Dengue fever is the most important mosquito-borne viral disease in Southeast Asia. Insecticides remain the most effective vector control approach for *Aedes* mosquitoes. Four main classes of insecticides are widely used for mosquito control: organochlorines, organophosphates, pyrethroids and carbamates. Here, we review the distribution of dengue fever from 2000 to 2020 and its associated mortality in Southeast Asian countries, and we gather evidence on the trend of insecticide resistance and its distribution in these countries since 2000, summarising the mechanisms involved. The prevalence of resistance to these insecticides is increasing in Southeast Asia, and the mechanisms of resistance are reported to be associated with target site mutations, metabolic detoxification, reduced penetration of insecticides* via* the mosquito cuticle and behavioural changes of mosquitoes. Continuous monitoring of the status of resistance and searching for alternative control measures will be critical for minimising any unpredicted outbreaks and improving public health. This review also provides improved insights into the specific use of insecticides for effective control of mosquitoes in these dengue endemic countries.

**Supplementary Information:**

The online version contains supplementary material available at 10.1186/s13071-021-04785-4.

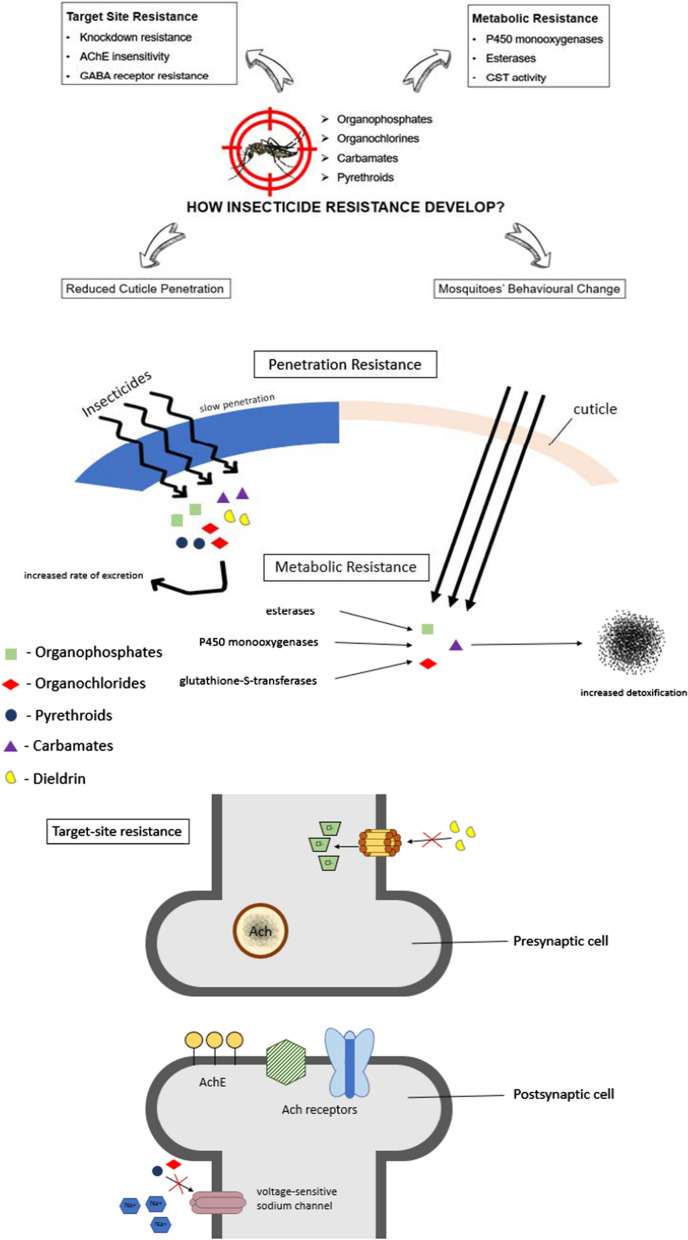

## Background

*Aedes* mosquitoes (Diptera, Culicidae) are the main vectors for several diseases associated with arboviruses, such as dengue, dengue haemorrhagic fever, dengue shock syndrome, yellow fever, chikungunya and Zika virus infection. There are two medically important species of *Aedes* mosquitoes that are associated with the transmission of dengue virus: *Aedes aegypti* (Linnaeus, 1762) and *Aedes albopictus* (Skuse, 1984). The adults of *Ae. aegypti* and *Ae. albopictus* are both black in colour but they can easily be differentiated by the pattern of white scales on their dorsal side of the thorax: *Ae aegypti* has two straight lines surrounded by curved lyre-shaped lines on the side while *Ae. albopictus* has a single broad line of white scales at the middle of the thorax [1]. The adult female *Aedes* mates, takes blood meals, lays 60–100 eggs in artificial and natural containers and can survive an average of 20–30 days. *Aedes *mosquitoes are considered to be day-time biters as they bite during dawn after sunrise and at dusk before sunset. Upon ingestion of dengue virus from an infected person, the virus will multiply in the salivary gland of the mosquito for 8–10 days (incubation period) prior to transmission to another person during subsequent blood meals. The flight range of *Aedes* mosquitoes is relatively short, in the range of 50 to 200 m from their breeding sites [1].

**Fig. 1:**
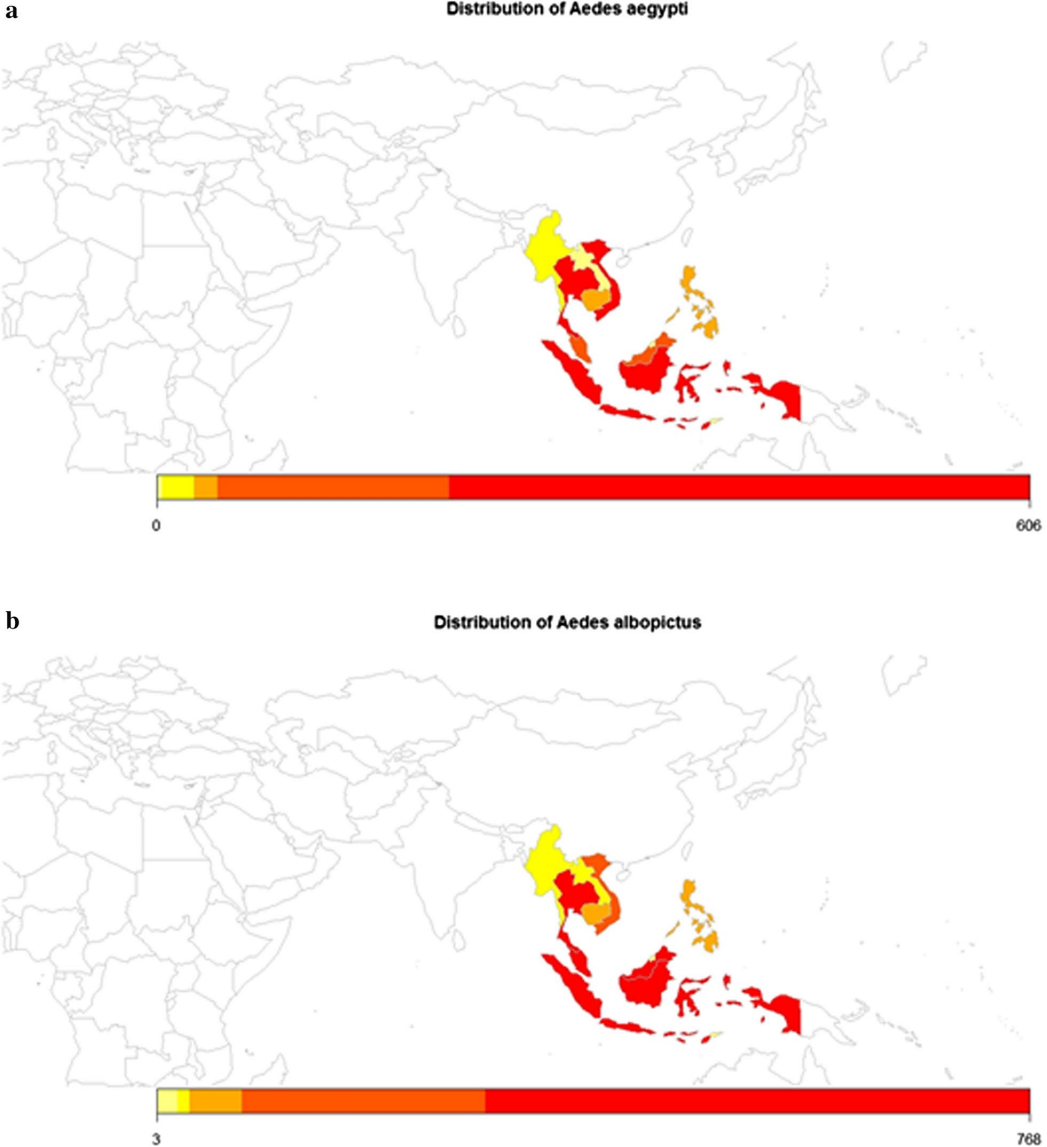
The occurrence of *Aedes aegypti* and *Aedes albopictus* in the Southeast Asia region [5]. Yellow - very low; light orange - low; dark orange - moderate and red - high occurrence

*Aedes aegypti* originated from Africa as a zoophilic tree-hole breeder (*Ae. aegypti formosus*) [2] and is domesticated or stays in close proximity to humans throughout the tropical and subtropical regions outside of Africa. This human-adapted species is hypothesised to have spread to the New World and Asia* via* increased global trade. *Aedes albopictus* (Skuse, 1894) is originally from Bengal, India and is indigenous to Southeast Asia [3]. It has spread to Africa, the Middle East, Europe, North and South Americas and Pacific Islands. To date, both *Ae. aegypti* and *Ae. albopictus* are widely distributed throughout the world, including Southeast Asia [4, 5] (Fig. 1).

Four antigenically distinct serotypes of dengue viruses (DENV-1, DENV-2, DENV-3 and DENV-4) can be transmitted to humans during the bite by an infected female *Aedes* mosquito (also known as horizontal transmission). Following the blood meal, the virus attaches to various cellular receptors and enters* via* cell-mediated endocytosis into midgut cells of the host. From the midgut, the viruses disseminate systematically* via* haemocoel or the body cavity to other secondary tissues, such as the salivary glands [6]. Natural vertical transmission of dengue viruses from the infected females to their offspring has also been reported in many dengue endemic countries [7].

**Fig. 2:**
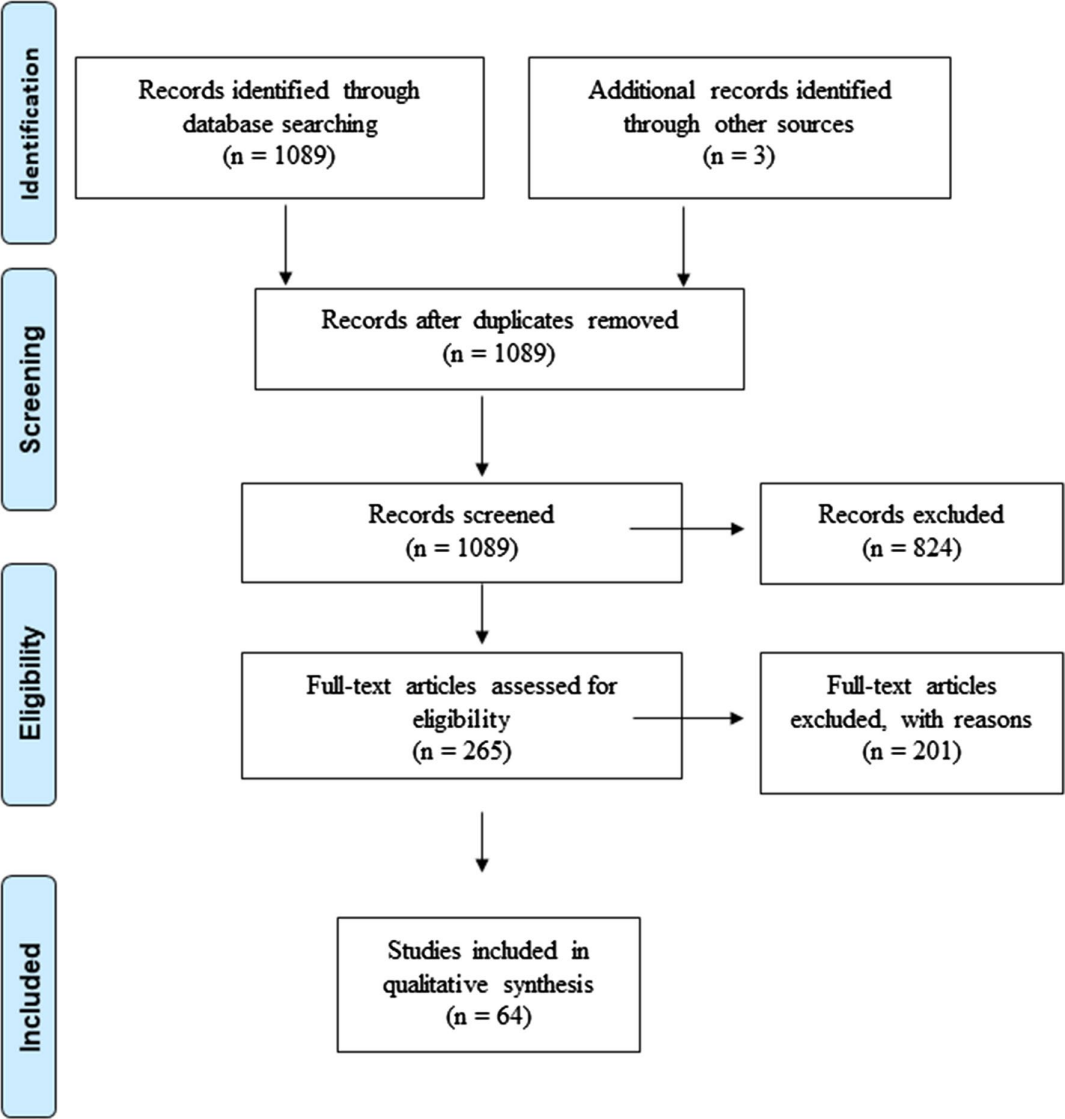
PRISMA (Preferred Reporting Items for Systematic reviews and Meta-Analyses) flow diagram of identification, screening and inclusion of studies included in this review [8]

In this paper, we review the distribution of dengue fever from 2000 to 2020 and its associated mortality in each Southeast Asian country. We also gather evidence on the trend of insecticide resistance and its distribution in these countries since 2000, summarising the mechanisms involved. To this end, we searched the PubMed (Medline), Google and Google Scholar databases for articles on insecticide resistance in dengue vectors in Southeast Asia, using the following search terms: ‘insecticide susceptible’ or ‘insecticide resist’ or ‘pyrethroid resist’ or ‘insecticide resistance’ and ‘Southeast Asia’ or ‘Asia, Southeastern’ and ‘dengue’. The search was limited to articles in English that had been published between 2000 and 2020 (Fig. 2).

## Dengue fever

**Fig. 3:**
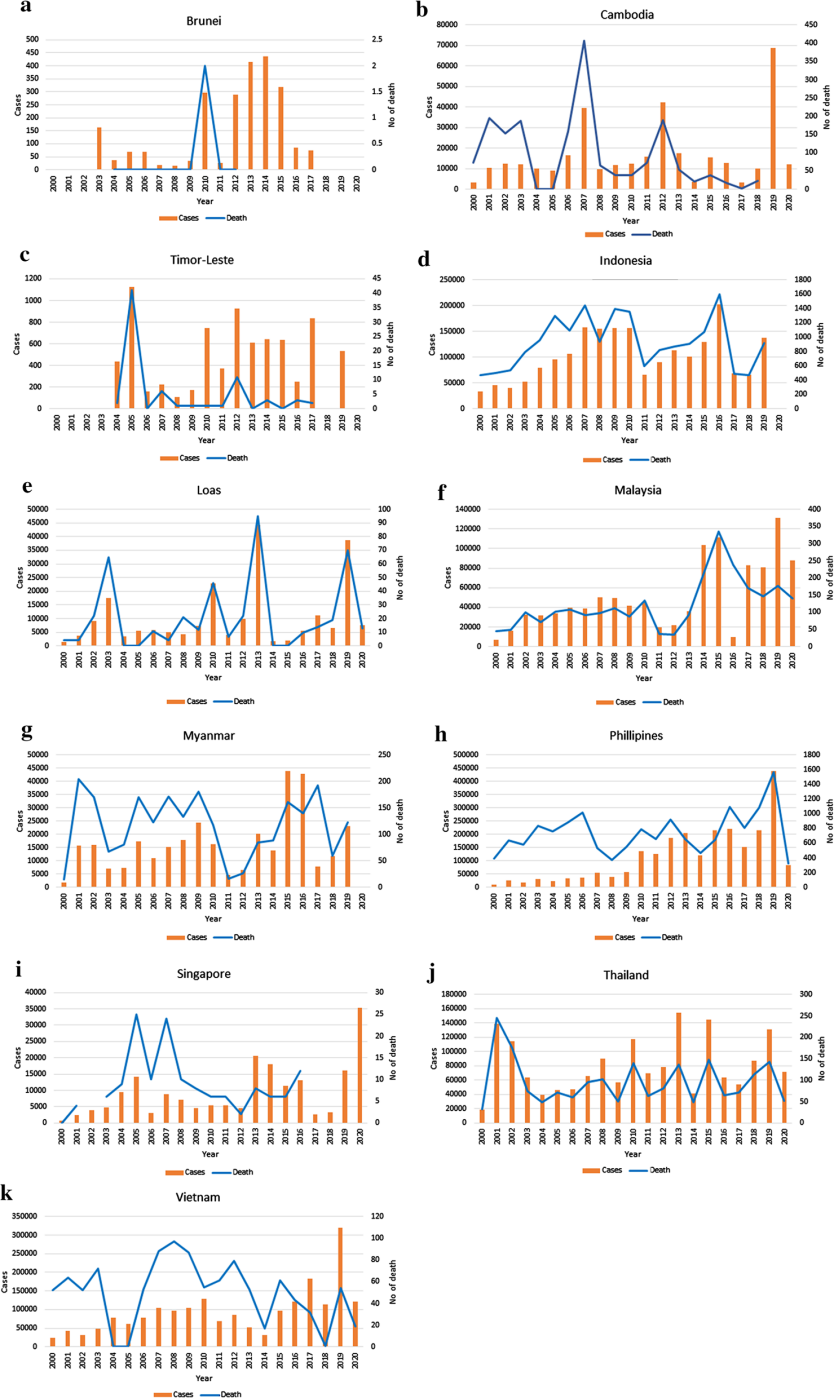
The number of dengue cases and its associated mortality in Southeast Asia from 2000 to 2019 [10–29]

Dengue virus can cause symptoms that range from a mild dengue fever to severe deadly dengue haemorrhagic fever and dengue shock syndrome. Annual estimates by Bhatt et al. [9] revealed that the number of dengue cases reported worldwide in 2010 was approximately 390 million, of which 96 million represent apparent dengue infections (dengue haemorrhagic fever or dengue shock syndrome). Asia contributed 67% (47–94 million infections) to this global disease burden [9]. Most countries in Southeast Asia experience frequent and cyclical epidemics of dengue throughout the year. The prevalence of dengue and its associated mortality for each Southeast Asia country are illustrated in Fig. 3 [10–29].

There is an additional 294 million inapparent infections (mild or asymptomatic) that are not detected by the current health surveillance system [9]. Those persons with inapparent dengue infection may not show any clinical manifestations of typical dengue infections or present with just mild illness that does not require a visit to a healthcare provider or hospitalisation. Hence, the inapparent dengue infection may not be captured as the burden of dengue infection. The prevalence of inapparent dengue is overwhelming and varies by geographical location, time and demography. In Malaysia, the seroprevalence of dengue has been reported to range from 28 to 92% [30, 31], with almost nine out of ten individuals who were dengue-seropositive not recalling having a previous dengue infection [31]. In one study, almost 10% and 70% of individuals without any history of dengue infection in Selangor, Malaysia tested positive for immunoglobulins M and G (IgM and IgG), respectively, against dengue virus, and ten out of 11 individuals with dengue viremia were asymptomatic [32]. Currently, the clinical significance of inapparent dengue infections remains undetermined, but it is highly suspected that inapparent dengue plays an important role in the maintenance of dengue transmission in the absence of an epidemic. Blood-feeding experiments with *Ae. aegypti* mosquitoes revealed that people with asymptomatic and pre-symptomatic DENV infections (low level of viremia) are capable of infecting mosquitoes [33]. In fact, DENV-infected people with no detectable symptoms or before the onset of symptoms are significantly more infectious to mosquitoes than people with symptomatic infections as DENV viraemic individuals without clinical symptoms may be exposed to more mosquitoes through their undisrupted daily routines than sick people. Furthermore, asymptomatic infections account for the bulk of DENV infections, thereby contributing significantly more to virus transmission to mosquitoes than previously recognised. Bosch et al. [34] revealed that people with asymptomatic infections are approximately 80% as infectious to mosquitoes as their symptomatic counterparts. The clinically inapparent infections may account for 84% of all dengue transmissions [34], with only 1% of DENV transmission attributable to people with clinically detected infections after they have developed symptoms.

## Insecticides as a control strategy of dengue

Due to the unavailability of an effective vaccine for dengue, one of the best approaches to control the spread of dengue is by managing the vector and its breeding sites. Various strategic approaches have been promoted to control mosquito vectors, including chemical control (indoor residual spraying, mass fogging, use of household insecticides), biological control (use of mosquito predators, release of specific genetic modified mosquitoes), source reduction and public education. Larvicidal chemicals, such as temephos and *Bacillus thuringiensis israelensis* (*Bti*), and adulticidal chemicals in ultra-low-volume sprays and fogging are widely used to control the spread of the disease. An estimated 2.5 million tons of pesticides are used annually [35]. There are four main classes of insecticides commonly used for vector control programmes: pyrethroids, organophosphates, organochlorines and carbamates.

### Organochlorines

Organochlorines (OCs) are chlorinated hydrocarbons that were developed in the early 1940s, and this category includes dieldrin, lindane, chlorobenziate, chlordane and the most popular chlorinated insecticide of all time, dichlorodiphenyltrichloroethane (DDT) [36, 37]. OCs are effective in controlling malaria, but they persist in the environment as a result of their high lipid solubility [38, 39]. They are subdivided into two subclasses, namely DDT-type chlorinated insecticides and chlorinated alicyclic insecticides, based on their distinct mechanisms and target sites [38]. DDT-type insecticides target the voltage-sensitive sodium channel (Vssc) in mosquitoes. Loughney et al. [40] described that the α-subunit of the sodium channel contains four homologous domains (I–IV), with each domain characterised by six transmembrane segments (S1–S6). Segments S1–S4 constitute the voltage-sensing domain whereas segments S5 and S6 form pore domains along with the intervening pore loop. The function of Vssc is to initiate and propagate action potentials in response to membrane depolarisation by opening and closing the channel [41]. DDT exerts its toxicity by impeding the sodium channels, hence retaining the conduction of sodium ions even after membrane repolarisation [42–44]. DDT acts mainly on the peripheral nervous system causing ‘DDT jitters’ where the muscles twitch throughout the body and the appendages. Exposure to DDT gradually leads to excitatory paralysis and subsequent death of the insects [44]. Chlorinated alicyclic insecticides, in comparison, bind at the γ-aminobutyric acid (GABA) molecule in the GABA chloride ionophore complex, resulting in hyper-excitation of the nervous system that subsequently leads to chlorine channel closure [45, 46].

### Organophosphates

Organophosphates (OPs), a phosphoric acid derivative, are the most toxic insecticides and detrimental to both mammals and insects. The most commonly used OPs are malathion, parathion, chlorpyriphos and diazinon. OPs were introduced in the 1960s to replace the usage of OCs with their many adverse effects and long persistence in the environment [39]. OPs interfere irreversibly with acetylcholinesterase (AChE) activity by phosphorylating its serine residues, resulting in hyper-excitation and disruption of neurotransmission in the central and peripheral nervous systems [37, 47]. This enzyme hydrolyses acetylcholine and causes repolarisation of basal plate in neuromuscular connections in preparation for the arrival of the new impulses [37]. Hence, the acute symptoms of poisoning with OP insecticides are muscle cramps, paralysis of respiratory muscles, convulsions and eventually death [36, 48].

### Carbamates

Carbamate insecticides are derivatives of carbamic acid. The carbamate insecticides, such as carbaryl, carbofuran, propoxur and aldicarb, exhibit similar effects as the organophosphorus insecticides by inhibiting cholinesterase activity [49]. Nonetheless, the toxic action of carbamates can be reversed whereas the action of OPs is irreversible. In addition, the toxicity of carbamates is rather short as the residue of carbamylated serine is less stable, where decarbamylation tends to split the carbamyl moiety from the enzyme [50].

### Pyrethroids

Pyrethroids are the synthetic analogues of natural insecticidal esters of chrysanthemum acid, called pyrethrins, which are categorised into types I and II based on their physical properties and toxicities. These insecticides have been widely used for the control of disease vectors for more than three decades [51]. The understanding of pyrethroids is complicated by two distinct intoxication syndromes. Type I pyrethroids (permethrin, tetramethrin, allethrin, phenothrin) lack an α-cyano group, and exposure causes tremor type syndrome by changing the conformation of the sodium channels for prolonged channel opening [51–53]. On the other hand, type II pyrethroids (cyfluthrin, cyhalothrin, deltamethrin, cypermethrin) possess an α-cyano-3-phenoxybenzyl moiety which produces choreoathetosis-salivation syndrome by modulating GABA levels and subsequently affect chlorine channels [51, 53, 54]. Type II pyrethroids have similar effects on sodium channels as type I pyrethroids, but with a lower amplitude of action potential. Type I pyrethroids give rise to repetitive discharges of sodium channels [38]. Previous studies have reported that the neurotoxicity of pyrethroids could be related to abnormal voltage-gated calcium regulation [52]. Certain pyrethroids, including cyfluthrin, cyhalothrin, cypermethrin, deltamethrin and permethrin, promote excessive calcium ion influx due to the reversed sodium–calcium exchange [51, 55].

## Mechanisms of insecticide resistance

**Fig. 4:**
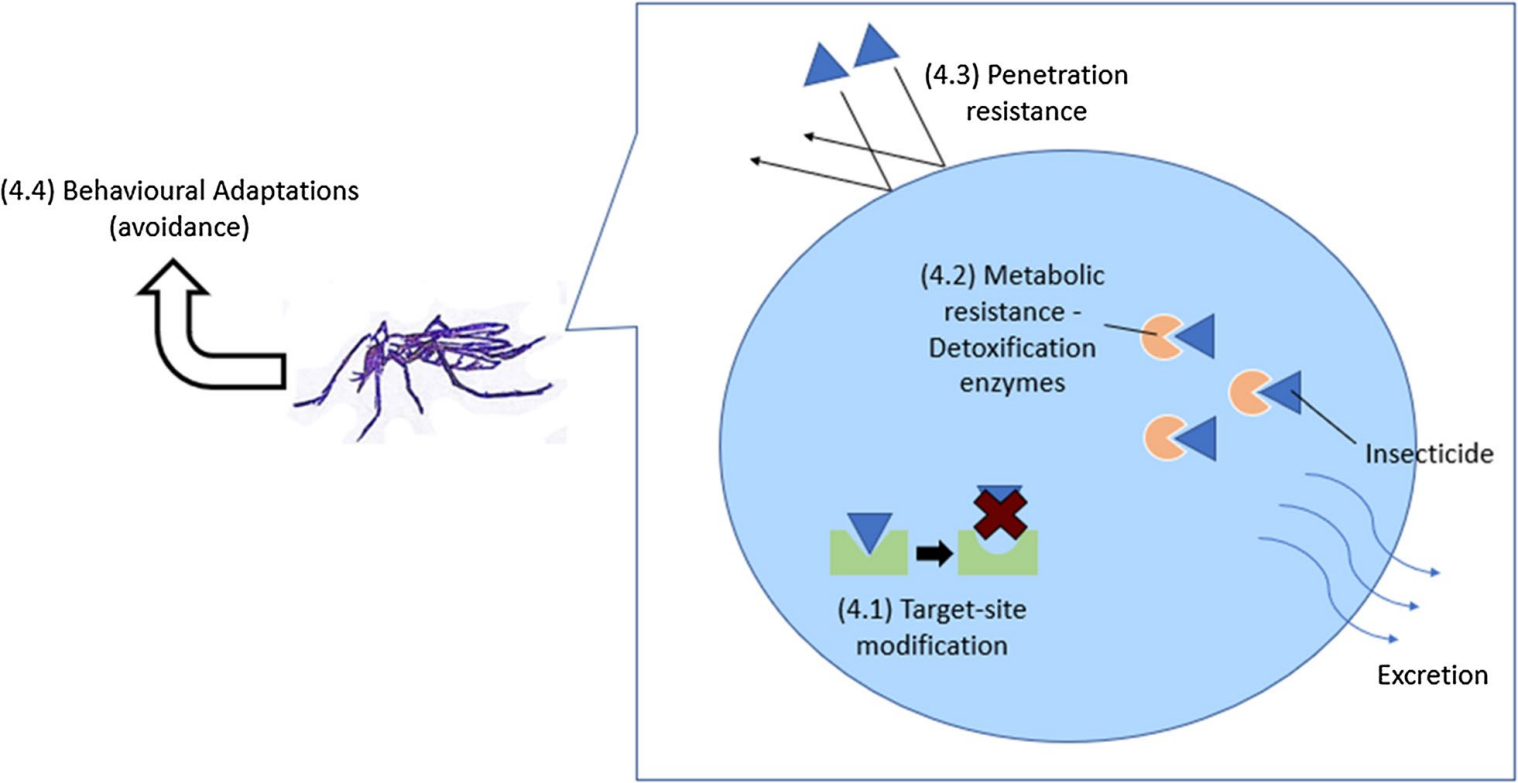
Mechanism of insecticide resistance i.e. target site resistance (4.1), metabolic resistance (4.2), penetration resistance (4.3) and behavioural adaptation (4.4)

Massive use of insecticide-based controls has contributed to the development of insecticide resistance, with increased challenges in eliminating *Aedes* mosquitoes and hence an increased risk of dengue transmission. The mechanism of insecticide resistance may include—but is not limited to—target site resistance, metabolic resistance, penetration resistance and behavioural adaptation (Fig. 4).

### Target site resistance

Target site resistance in mosquitoes is inferred when the targeted site for the action of insecticides is genetically modified, thus limiting its interaction with neurotoxins and consequently eliminating the insecticidal effects. These modifications may include the Vssc mutation, insensitivity of synaptic acetylcholinesterase (AChE1) and mutation in the GABA receptor [56].

#### Knockdown resistance

Knockdown resistance (*kdr*, also known as the *Vssc* mutation) is the major mechanism of sodium channel insensitivity to both DDT and pyrethroids [57, 58]. Most *Vssc* mutations are located at domains IS6, IIS6 and IIIS6.* kdr* caused by point mutations of the target site, with the substitution of leucine (L) by phenylalanine (F), histidine (H) or serine (S) in *Vssc* in IIS6 at codon 1014, has been reported in mosquitoes of genera *Anopheles* and *Culex* [59]. In *Ae. aegypti* mosquitoes, various pyrethroids/DDT resistance-associated mutations (G923V, L982W, I1011M/V, V410, T1520I, S989P, F1534S/L/C, D1763Y, V1016G/I) have been documented [60–64]. For example, the T1520L mutation was identified in *Ae. aegypti* populations from India and the V1016G mutation in *Ae. aegypti* populations from Malaysia and Thailand [65–67]. In addition, co-occurrence of multiple *kdr* mutations has been commonly associated with higher levels of phenotypic resistance to DDT and pyrethroids [68, 69]. Co-existing mutations of V1016G/S989P have been reported in Malaysia, Thailand and other Southeast Asia regions, and co-existing mutations of V1016G/F1534C were found in Singapore in *Ae. aegypti* populations [66, 70, 71]. *Vssc* mutations can also confer cross-resistance between DDT and pyrethroids due to reduced sensitivity of the nervous system [72, 73]. It is remarkable that *Vssc* mutations have expanded greatly over the last three decades (Table 1) but that the specific mutations that confer the resistance have yet to be identified.

**Table 1:** Knockdown resistance (Vssc) mutations that have been detected in different mosquito populations

Mutation	Transmembrane domain	Mosquito	References
G923V	II	*Aedes aegypti*	[61]
L982W	II	*Ae. aegypti*	[61]
I1011M	II	*Ae. aegypti*	[61]
I1011V	II	*Ae. aegypti*	[74]
V410L	I	*Ae. aegypti*	[75]
T1520I	III	*Ae. aegypti*	[65]
S989P	II	*Ae. aegypti*	[63]
F1534S	III	*Ae. albopictus*	[76]
F1534L	III	*Ae. albopictus*	[76]
F1534C	III	*Ae. albopictus*, *Ae. aegypti*	[75, 77]
D1763Y	IV	*Ae. aegypti*	[78]
V1016G	II	*Ae. aegypti*	[63]
V1016I	II	*Ae. aegypti*	[79]

*Vssc *Voltage-sensitive sodium channel

#### Synaptic AChE insensitivity

Acetylcholinesterase is the primary target of OP and carbamate insecticides which block the transmission of nerve impulses at cholinergic synapses. An understanding of OP and carbamate resistance is demonstrated by the insensitivity of AChE subsequent to amino acid substitutions at the target gene, acetylcholinesterase 1/2 (*ace-1* or *ace-2*) [80, 81]. To date, only three amino acid substitutions have been described in different mosquitoes species: the substitution of glycine to serine at codon 119, of phenylalanine to valine at codon 290 and of phenylalanine to tryptophan at codon 331 [80–83] (Table 2). For example, the G119S mutation has been extensively studied in *Culex pipiens* and *Anopheles gambiae*, but the involvement of the *ace* gene on insensitive AChE in certain mosquito species, including *Ae. aegypti* and *Anopheles stephensi*, remains to be identified. G119S substitution results in steric hindrance, which reduces substrate or inhibitor binding, whereas F290V and F331W both modify the stabilisation process [84]. The G119S mutation occurs in the oxyanion hole of acetylcholinesterase 1 which aids in substrates trafficking [84, 85]. The F290V mutation involves substrate specificity and the F331W mutation has been denoted as being involved in substrate guidance and binding [80, 86, 87].

**Table 2:** Acetylcholinesterase mutations observed in different mosquito populations

Mutation	Gene	Mosquitos	References
G119S	*ace-1*	*Culex pipiens*, *Culex vishnui, Anopheles gambiae* and *Anopheles albimanus*	[80–82]
F290V	*ace-1*	*C. pipiens*	[83]
F331W	*ace-2*	*Culex tritaeniorhynchus*	[82]

*ace-1*/*ace-2* Acetylcholinesterase 1/2

#### GABA receptor resistance

The GABA receptor is encoded by the resistance to dieldrin (*RDL*) gene involved in neuronal signalling [88]. The RDL receptor is a member of Cys-loop ligand-gated ion channel superfamily with a N-terminal extracellular domain for GABA binding. This receptor contains five subunits, with each subunit having an extracellular cysteine loop and four transmembrane domains (M1–M4) [89]. RDL is the target of various insecticides, such as cyclodiene, fipronil and pyrethroids, where its function is influenced by the post-translational modifications [88, 90]. Several findings suggest that the complexity of RDL receptors is formed by alternative splicing at axons 3 and 6 and RNA editing [91]. Here, adenosine residues are replaced with inosine in transmembrane segment M2 of* RDL* through the action of adenosine deaminases, resulting in the removal of the amine group and subsequently leading to formation of different isoforms. Studies by Taylor-Wells et al. [88, 92] have documented the identification of species-specific RNA A-to-I editing sites in the RDL of insecticide-resistant mosquitoes. For example, an alanine to serine or glycine substitution at position 296 is found in *Ae. aegypti* [56] and *Ae. albopictus* [93]. This mutation does not affect insecticide sensitivity but it does reduce the fitness as a result of the A296G substitution which greatly impacts neuronal signalling [88].

### Metabolic resistance

Resistant strains detoxify the toxins/insecticides much better than susceptible mosquitoes due to the overexpression of or conformation change in enzymes subsequent to point mutations in* cis*/*trans* loci of the enzymes [94]. Metabolic detoxification is usually associated with three major enzymatic activities, such as cytochrome P450 monooxygenases, esterases and glutathione S-transferases (GST) activity [56, 60, 95].

#### P450 monooxygenases

Cytochrome P450 (CYP) monooxygenases are one of the primary resistance mechanisms of pyrethroids in mosquitoes. CYP is a hydrophobic, heme-containing enzyme which metabolises a number of exogenous and endogenous compounds* via* oxidation in the presence of NADPH-CYP reductase (CPR) and occasionally cytochrome b5 [56, 96]. Overexpression of CYPs or mutation at an open reading frame of CYPs have been reported in insecticide-resistant mosquitoes [97, 98]. The details of the molecular mechanisms are poorly characterised due to the presence of large number of CYPs. Table 3 summarises the known overexpressed CYPs and their associated insecticide resistance in *Aedes* mosquitoes. In *Ae. aegypti*, CYP6Z8 plays a pivotal role in pyrethroid clearance* via* carboxyesterase-mediated hydrolysis that generates 3-phenoxybenzyl alcohol (PBAlc) and 3-phenoxybenzaldehyde (PBAld), and finally 3-phenoxybenzoic acid (PBAcid), all with lower toxicity to the mosquitoes as compared with intact pyrethroids [105, 108, 109].

**Table 3:** Increased cytochrome P450 expression in various mosquito populations against insecticides

Mosquitos	CYPs	Stage	Insecticide	References
*Ae. aegypti*	CYP4H28	Larvae	Temephos	[99]
*Ae. aegypti*	CYP6AH1	Larvae	Temephos	[99]
*Ae. aegypti*	CYP6CB1	Adults	Permethrin	[100]
*Ae. aegypti*	CYP6F3	Larvae	Permethrin	[101]
*Ae. aegypti*	CYP6M6	Larvae and adults	Deltamethrin	[102, 103]
*Ae. aegypti*	CYP6M10	Larvae	Permethrin	[101]
*Ae. aegypti*	CYP6M11	Larvae	Permethrin, Temephos	[102, 103]
*Ae. aegypti*	CYP6N12		Temephos	[104]
*Ae. aegypti*	CYP6Z6	Larvae and adults	Deltamethrin	[102, 103]
*Ae. aegypti*	CYP6Z8	Larvae and adults	Deltamethrin, Temephos	[99, 102, 103, 105]
*Ae. aegypti*	CYP9J10	Adults	Permethrin	[100]
*Ae. aegypti*	CYP9J19	Adults	Permethrin	[100]
*Ae. aegypti*	CYP9J22	Larvae and adults	Deltamethrin	[102, 103]
*Ae. aegypti*	CYP9J23	Larvae and adults	Deltamethrin	[102]
*Ae. aegypti*	CYP9J24	Adults	Permethrin	[100, 106]
*Ae. aegypti*	CYP9J26	Adults	Permethrin	[100, 106]
*Ae. aegypti*	CYP9J27	Adults	Permethrin	[100]
*Ae. aegypti*	CYP9J28	Adults	Permethrin	[101]
*Ae. aegypti*	CYP9J32	Adults	Deltamethrin, Permethrin	[100, 106]
*Ae. aegypti*	CYP12F6	Adults	Permethrin	[100]
*Ae. aegypti*	CYP304C1	Adults	Permethrin	[100]
*Ae. Albopictus*	CYP6AG6	Adults	Deltamethrin, Permethrin	[107]
*Ae. albopictus*	CYP6N3	Adults	Bendiocarb	[107]
*Ae. albopictus*	CYP6P12	Adults	Deltamethrin, Permethrin	[107]
*Ae. albopictus*	CYP6Z6	Adults	Deltamethrin, Permethrin	[107]

#### Esterases

Esterase-mediated resistance to OPs, pyrethroids and carbamates have been studied extensively in *Culex* mosquitoes. Esterases act by rapid binding or slow turning, i.e. sequestration, to prevent the interactions between insecticides and AChEs [110]. Furthermore, increased production of esterases was reported to be closely related with amplification of the esterase alpha 2 genes [111, 112]. Two genes, *estα2* and *estβ2*, are involved in detoxifying carboxylester hydrolase expression and esterase overproduction [113]. Both of them hydrolyse the ester bonds to produce alcohols and acids as metabolites* via* a two-step reaction which involves nucleophilic attack of the serine residue on the carbonyl carbon of the ester bond, followed by a second nucleophilic attack by water molecules to replace the acyl group, resulting in the release of the free active enzyme and acidic moiety of the carboxylic ester [114, 115]. These two loci are differentially transcribed with average ratios of *estβ2* over *estα2* at 10:1 and 15.9:1, respectively, in all resistant *Culex* mosquitoes [116]. Enhanced esterase activities in insecticide-resistant *Ae. aegypti* has been reported but the genes involved are yet to be identified [117].

#### Glutathione* S*-transferase activity

Glutathione* S*-transferases belong to a large and multifunctional enzyme family participating in detoxification of xenobiotics, such as insecticides. They are classified into two ubiquitously distant classes: microsomal and cytosolic GSTs, respectively. Microsomal GST has a trimeric structure and its associated mechanism of insecticide resistance has yet to be elucidated. On the other hand, insect cytosolic GSTs are dimeric proteins comprising two subunits of 24–28 kDa each [56, 118]. Another group of GSTs (kappa GSTs) are located in mammalian mitochondria and peroxisomes, but this class of GSTs is absent from insects [119, 120]. Hence, only cytosolic GSTs have been implicated in insecticide resistance to date. A total of 26 GST genes have been reported in *Ae. Aegypti*, of which two can splice alternatively, which results in a total of 29 transcripts for cytosolic GSTs [121]. Mechanisms of GST-mediated insecticide resistance have been depicted as occurring either directly* via* the GST conjugation reaction (phase I) or* via* metabolism of secondary products by other detoxifying enzymes, such as P450 (phase II) [122]. In phase I metabolism, GSTs catalyse the nucleophilic attack of the thiol group of reduced glutathione located in the electrophilic centre of lipophilic compounds, including OPs and pyrethroids, causing increased water solubility and excretion by the cells [118, 122, 123]. Another GST-based detoxification occurs when GSTs serve as a co-factor of dehydrochlorination by removing a hydrogen atom from its substrate [124]. This reaction has been implicated in resistance to DDT. In addition, certain GSTs confer resistance by passive binding or detoxifying lipid peroxidases and reactive oxygen species subsequent to the induction of oxidative stress [117, 119, 123, 124].

To date, there are at least six classes of GSTs in *Ae. aegypti* (theta, sigma, zeta, omega, delta and epsilon) [125]. GSTE2 in the Epsilon class is overexpressed in DDT-permethrin-resistant *Ae. aegypti* [126].

### Penetration resistance

Penetration resistance occurs when barriers develop at the outer cuticle of mosquitoes, resulting in slow absorption of insecticides into their bodies. Likewise, resistant mosquitoes absorb toxins at a much slower rate than susceptible strains. Reduced penetration in turn provides more time for detoxification by facilitating the action of metabolic enzymes. Thus, this cuticular resistance is usually involved in cross-resistance to multiple insecticides due to their lipophilic property [127]. Overexpression of CYP enzymes, including CYP4G16 and CYP4G17, facilitates the deposition of cuticular hydrocarbons in the epicuticle of the pyrethroid-resistant mosquitoes, such as *Ae. aegypti* [128, 129]. Hence, large amounts of cuticular hydrocarbons are formed and deposited on top of cuticle that function as a waterproofed layer conferring desiccation resistance. This mechanism on lipid transport and epicuticular deposition is the least understood of all the mechanisms described herein and should be further investigated to define its role in insecticide resistance.

### Behavioural adaptation

Mosquitoes can reduce or prevent negative consequences of insecticides through adaptations. Behavioural resistance is generally categorised into temporal, spatial and trophic avoidance whereby the mosquitoes escape from coming into contact with insecticides. The term ‘temporal avoidance’ involves a mosquito reducing its risk of exposure by mismatch to the timing when insecticides are employed whereas ‘spatial avoidance’ involves the mosquitoes moving away from the insecticide-treated areas [130, 131]. Mosquitoes apply trophic avoidance by avoiding feeding on hosts in areas where insecticides are extensively used [130, 131]. Therefore, many researchers have hypothesised that behavioural changes may also be considered as a mechanism of resistance. In addition, behavioural tolerance evolves when mosquitoes that are unable to escape from the exposure develop tolerance through limiting their fitness loss. Mosquitoes can alter their behaviours by increasing their current reproductive effort, such as adjusting their egg production patterns, reducing their energy expenditure and maximising their nutrient uptake [132]. This proposed theoretical classification of behavioural resistance is poorly deciphered and the fitness costs are yet to be quantified.

## Prevalence of insecticide resistance in Southeast Asia

Various insecticides have been used worldwide for the control of the vector-borne diseases, including dengue. However, the effectiveness of this measure in controlling *Aedes* mosquitoes needs to be considered in light of the increasing trends of resistance towards different insecticides at different geographical locations. Studies on insecticide resistance and its prevalence in *Aedes* mosquitoes may be limited or insufficient in certain Southeast Asian countries. The summary of the studies on the prevalence of insecticide resistance in all of the Southeast Asian countries included herein is provided in Additional file 1: Table S1.

### Cambodia

Dengue fever is a major public health issue in Cambodia, with an estimated 185,000 cases in that country annually [133, 134]. The application of large amounts of insecticides was initially effective in decreasing the number of dengue cases, but despite the little information currently available, the incidence of insecticide resistance can be seen to be increasing. In Cambodia, temephos is used to control larvae whereas deltamethrin and permethrin are used as adulticides. Recently, resistance of Cambodian *Ae. aegypti* populations to temephos has been appearing in Phnom Penh, Battambang and Kampong Cham, where the resistance ratios were reported to be 5.3, 33.6 and 8.4 in urban areas and 5.3, 13.0 and 11.2 in rural areas, respectively (a ratio > 5 is an indication of resistance) [134, 135]. Fortunately, *Ae. aegypti* larvae remain susceptible to temephos in both urban and rural areas in Siem Reap [133]. Boyer et al*.* [133] reported strong resistance to permethrin, with an average mortality rate of 2.22%, and a lower resistance to deltamethrin, with a mortality percentage of < 90%. The V1016G, S989 and C1534C mutations have also been detected in Cambodia in *Ae. aegypti* populations at a high frequency [136, 137].

### Indonesia

In 2018, 65,602 cases of dengue fever were reported, of which 467 were fatal [29]. Low mortality rates of *Ae. aegypti* larvae (0–1.33%) were observed in several cities against malathion insecticide, possibly due to the massive use of malathion in fogging for past three decades [138]. *Aedes aegypti* larvae from Surabaya, Indonesia were reported to be resistant to temephos, with moderate mortality rates ranging from 16 to 60% [138, 139]. Knockdown resistance against pyrethroid insecticides was associated with V1023G and S996P mutations in *Ae. aegypti* larvae in Yogyakarta, and with the S989P and V1016G mutations in Denpasar, Bali [140, 141]. Adult *Ae. aegypti* that were resistant to pyrethroids carried *Vssc* gene mutations. Three point mutations (V1016G, F1534C and S989P) were associated with pyrethroid resistance [137, 142, 143]. Permethrin resistant *Ae. aegypti* from Makassar, Sulawesi, Indonesia were associated with the V1016G mutation [144]. *Ae. aegypti* populations obtained from Padang Jati and Gunung Pangilun were resistant to temephos. A point mutation at *ace-1* of these temephos-resistant *Ae. Aegypti* strains collected from Padang did not have the G119S substitution, but instead had the T506T substitution, a silent mutation [145].

Other than target site resistance, the mechanism of action could also involve metabolic detoxification. Increased levels of detoxifying enzymes, such as GSTs, oxidases and esterases, play important roles in conferring resistance to DDT, malathion, temephos or pyrethroids in mosquitoes collected from Bogor, Garut, Sumedang, Tasikmalaya or Sumerang, Indonesia [146]. For example, exposure of Sumedang mosquito populations to permethrin resulted in a 17-fold elevation of esterase activities and a fourfold elevation of mixed function oxidases [146]. These mosquitoes were significantly associated with the V1016G and S989P mutations [147]. Mosquitoes from Denpasar, Mataram, Kuningan, Padang, Samarinda and Sumba Timur were resistant to d-allethrin, transfluthrin and metofluthrin of the mosquito coils [148]. Nevertheless, susceptible strains were detected in Pontianak, Dompu and Manggarai Barat [148].

### Laos

Dengue is re-emerging in Laos, with several reported outbreaks between 2013 and 2017 in both rural and urban areas. The number of cases annually range from 2000 to 20,000, of which approximately ten are fatal. OCs such as DDT were used for vector control and agriculture in Laos from the 1950s until they were banned in 1989. The larvicide temephos, an OP, was first used to treat water in containers during the dengue outbreak in 1987. Malathion was then introduced in the 1990s for thermal fogging, followed by the use of pyrethroids (deltamethrin and permethrin) from early 2000s onwards.

Tangena et al*.* [149] reported that all *Ae. albopictus* collected from the capital city Vientiane and Luang Prabang province were resistant to DDT (27–90% mortality) and malathion (20–86% mortality) and susceptible to deltamethrin and permethrin (100% mortality), with the exception of one population from Kao-gnot, Vientiane City which was suspected of being resistant to permethrin (96% mortality). *Ae. albopictus* larvae were highly resistant to DDT (3–44%), and showed resistance to temephos in Luang Prabang (Huayhoy village, 74% mortality) and Vientiane City (Suanmone and Oudomphon, 42 and 87% mortality, respectively) [149].

Marcombe et al*.* [150] investigated insecticide resistance in *Ae. aegypti* populations collected from 11 villages located in five provinces in Laos to larvicides and adulticides used in Laos. All *Ae. aegypti* larvae collected showed moderate to strong resistance to temephos, deltamethrin, permethrin and DDT. Similarly, the adult mosquitoes collected from most of the villages were highly resistant to DDT, permethrin and malathion but susceptible to deltamethrin. All resistant adult mosquitoes showed significant elevated CYP monooxygenases, GST and carboxylesterases. Two* kdr* mutations at V1016G and F1534C were detected in these populations, and a higher frequency of the F1543C *kdr* mutation (> 0.6) and low frequency of V1016G mutation (< 0.36) were found in resistant strains [150].

Marcombe et al. [151] conducted a simulated field trial of temephos, *Bti*, diflubenzuron, pyriproxyfen and spinosad using an established *Ae. aegypti* colony (IPL strain) obtained from wild, field-caught mosquito larvae collected using ovitraps placed at the Institut Pasteur du Laos (IPL), Vientiane in Kao-gnot village. This wild IPL field strain was susceptible to *Bti*, diflubenzuron and pyriproxyfen (resistance ratio [RR] = 1) but showed moderate resistance to temephos and spinosad (RR < 5). These results suggest that *Bti*, diflubenzuron and pyriproxyfen may be used as alternative larvicides for dengue vector control in water-storage containers in Laos at places with temephos-resistant mosquito populations.

### Malaysia

As of 12 December 2020, a cumulative 88,074 dengue cases had been reported in Malaysia, which marks a drastic decrease compared with the 124,777 cases for the same period in 2019 [15]. Studies have been conducted in all 13 states to evaluate the status of insecticide resistance in the dengue vectors and the associated resistance mechanisms. Rosilawati et al*.* [152] conducted a comprehensive study on 12 dengue hotspots across five states in Peninsular Malaysia and revealed that 75% of the collected *Ae. aegypti* mosquitoes were resistant to permethrin. In particular, *Ae. aeygpti* from Bandar Baru Bangi (S15) exhibited higher knockdown rate of 600-folds compared with laboratory strains [152]. Rosilawati et al*.* [152] extended their study with another three dengue-endemic localities and characterised the resistance mechanisms in *Ae. aegypti*. All three field-collected strains exhibited strong resistance to pyrethroids with complete absence of mortality but were highly susceptible to OPs. Similarly, *Ae. aegypti* larvae collected in Selangor and Penang also showed same resistant patterns [153–155]. Other than pyrethroids, *Ae. aegypti* populations were also resistant to DDT and carbamate bendiocarb. Resistance profiles were associated with *kdr* mutations in Malaysian *Ae. aeygpti* populations. Most of these strains harboured the F1534C, V1016G and V1023G substitution alone or combination mutations of V1023G and S996P [66, 153, 156].

On the other hand, *Ae. albopictus* populations, secondary vectors of dengue, were found to be mostly fully susceptible to pyrethroids, with Kuala Lumpur strains showing a moderate tolerance to deltamethrin and permethrin [66]. In that study, variation in the mortality rates of *Ae. albopictus* to DDT, bendiocarb, dieldrin and malathion in several states was reported and both *Aedes* species exhibited elevated levels of CYP and oxidase enzymes [66]. Ishak et al. [107] reported overexpression of *CYP6P4* in *Ae. albopictus* and *CYP6P12* in *Ae. aegypti* as being associated with pyrethroid resistance whereas *CYP6N3* was observed across DDT- and carbamate-resistant *Ae. albopictus* populations. Several CYP genes, including *CYP9J27*, *CYP9J26*, *CYP9J28*, *CYP9M6* and *CYP6CB1*, were found to be overexpressed in pyrethroid-resistant *Ae. aegypti* [157]. Overexpression of cuticular protein genes, which results in cuticle thickening, was associated with reduced penetration of pyrethroid in *Ae. albopictus* populations [107].

Research on insecticide resistance in East Malaysia is limited. Larvicide resistance in *Ae. albopictus* was reported in Sabah, particularly on the West Coast and in Kudat were strong resistance to DDT and malathion with complete survival, and to tempehos and bromophos with mortality rates ranging from 0 to 93.33% were observed [158]. Adult *Ae. albopictus* populations were susceptible to pyrethroids but displayed moderate resistance against the other three classes of insecticides in Sabah [159]. The prevalence of *Ae. albopictus* populations in Sabah could be explained by its geographical landscape of tropical rainforests, which is a favourable habitat for *Ae. albopictus*.

### Myanmar

The Ministry of Health and Sports reported 4121 cases of dengue fever with 32 deaths across Myanmar as of 11 July 2020 [26]. A small number of studies on the mechanisms of pyrethroid resistance in *Ae. aegypti* were reported in Myanmar. F1534C mutations were detected in permethrin-resistant *Ae. aegypti* in Yangon City [136]. Single point mutations of V1016G and S989P together with the co-occurrence of *kdr* mutations were observed in pyrethroid-resistant *Ae. aegypti* [160]. Three patterns of co-occurrence were observed, including V1016G/F1534C, V1016G/S989P and V1016G/F1534C/S989P, at varying frequencies of 2.9, 65.7 and 0.98%, respectively [160]. A genotyping study also revealed the presence of the wildtype VV/FF, double homozygous alleles (GG/CC), VG/CC and GG/FC derived from the F1534C and V1016G mutations in Yangon City [137]. DDT resistance, with less than 5% of mortality, was reported in *Aedes* mosquitoes collected at seven townships in Yangon City where 1.2 metric tons of DDT had been employed for dengue fever control until it was banned in 2003 [161].

### Philippines

In the Philippines, 420,000 dengue cases were reported in 2019, of which 1565 were fatal, which is double the number of cases reported the previous year for the same period [15]. *Aedes aegypti* collected from Mandaluyong City were only susceptible to malathion but resistant to all other insecticides [162]. To date, there is no report on insecticide resistance for both larvae and adults of *Aedes* mosquitoes in Philippines.

### Singapore

The National Environment Agency of Singapore disclosed a total of 701 dengue cases transmitted by *Aedes* mosquitoes as of 2 February 2021, which twofold lower than the number of cases during same period of the previous year [22]. *Ae. aegypti* mosquitoes in Singapore were reported to be resistant to permethrin and cypermethrin decades ago [163]. Similar to other countries, DDT and pyrethroid resistance among *Ae. aegypti* larvae and adult mosquitoes has been reported in Singapore [164, 165]. *Vssc* mutations were detected in pyrethroid- and DDT-resistant *Ae. aegypti* carrying the V1016G, F1534C and F1269C substitutions [71, 128], with the G1016 alleles contributing more significantly to the target site insensitivity than the C1534 alleles [128]. A combination of V101G, S989P and F1534C mutations modified the sensitivity of Vssc channels to deltamethrin and permethrin by 90- and 1100-fold, respectively [166]. A correlation between *CYP* genes, i.e. overexpression of *CYP6BB2*, *CYP9M6*, *CYP9M4*, *CYP9M5*, *CYP4C50*, *CYP6Z7*, *CYP6Z8* and *CYP6F3*, and permethrin resistance was detected [128]. Elevated esterase and GST levels may play an important role in pyrethroid and DDT resistance [164, 165]. F1534C alleles were detected in permethrin-resistant *Ae. albopictus* [167]. An association between high levels of mixed function oxidase and permethrin resistance has also been detected in *Ae. albopictus* populations [163].

### Thailand

Thailand reported 129,906 dengue cases in 2019 [17]. The susceptibility status and the resistance mechanisms of mosquitoes in Thailand are the most well-studied among the nations of Southeast Asia. In recent years, the tremendous use of all four classes of insecticides has resulted in an irreversible consequence, namely the development of insecticide-resistant mosquito strains, with *Ae. aegypti* populations in Thailand reported to be resistant to a wide range of insecticides, including deltamethrin, permethrin, fenitrothion, temephos, propoxur, DDT, cyfluthrin and alpha-cypermethrin [67, 168–170]. The *Vssc* mutation is the major mechanism of pyrethroid resistance, in which V1016G was detected to be associated with the S989P and F1534C substitution in the homozygous form [136, 166]. Additionally, triple heterozygous P989, G1016 and C1534 mutants were detected in deltamethrin-resistant mosquitoes [137, 171]. Another mutation, F1552C, was also detected in permethrin-resistant strains in several provinces of Thailand, including Chiang Mai, Song Khla and Ubon Rachathanee [172]. Metabolic detoxification was also found to be involved in insecticide resistance with an increased expression of monooxygenases (*CYP9J32*, *CYP6Z8*, *CYP9M9*, *CYP6AH1*, *CYP4H28*), GSTs (*GSTE2*) and carboxylesterases (*CCEAE3A*, *CCEAE4A* and *CCEAE6A*) detected in resistant *Ae. aegypti* [99, 106, 173, 174]. *Ae. albopictus* samples collected from Pong Nom Ron showed high resistance to all five pyrethroids, with mortality rates ranging from 34.4 to 68.6%. Rayong strains also showed resistant to permethrin with a 51% mortality rate [175].

### Timor-Leste

Timor-Leste reported 837 dengue cases in 2017, of which two were fatal [24]. Insecticide resistance resulting in ineffectiveness of dengue elimination was only reported once, in 2015. *Ae. aegypti* populations from Dili were found to be resistant to permethrin, lambda cyhalothrin and resmethrin in association with the overexpression of esterases [176].

### Vietnam

Dengue infections have increased substantially in Vietnam with 121,398 reported cases and 19 deaths as of 29 November 2020 (*vs* 314,468 cases and 54 deaths in 2019) [15]. The resistance of *Ae. aegypti* to DDT and pyrethroids was first reported in 1999 [177]. *kdr* mutations and overexpression of CYP enzymes in resistant *Ae. aegypti* populations were observed. *Ae. aegypti* resistant to permethrin has been reported in several provinces, including Nha Trang, Hanoi, Ho Chi Minh, Kien Giang, Dong Nai and Dak Lak, with mortality rates ranging from 3.03 to 52.25% [178]. In addition, *Ae. aegypti* mosquitoes were found to be resistant to lambda-cypermethrin, cyfluthrin, etofenprox, DDT and alpha-cypermethrin [178, 179]. Several point mutations (V1016G, V1016I and F1269C) were detected [178, 180]. V1016G mutations were also detected in permethrin-resistant *Ae. albopictus* populations from Hanoi [167].

### Brunei

To date, there is no or limited access to dengue data and resistance for Brunei.

## Conclusions and future perspectives

Although insecticides were once effective in controlling mosquito-borne diseases, the increasing trends of mosquito-borne diseases may indicate an increasing resistance to or ineffectiveness of insecticides in controlling the transmission of the diseases. Furthermore, insecticides may also significantly influence the environment and ecosystems. It may be wise to revisit the concept of using chemical insecticides for controlling or eliminating mosquitoes and hence disease transmission. Long-lasting insecticide nets (LLINs) and indoor residual spraying, the use of which has been implemented as public health intervention tools for mosquito control, now require more diversified products due to the overwhelming development of insecticide resistance among mosquito populations. Biological control strategies which target different stages of the mosquito life-cycle, such as the use of numerous copepods, including *Mesocyclops longisetus* and *M. thermocyclopoides* which prey on the young mosquito instars, could be an alternative control strategy[181, 182]. In Vietnam, copepod biocontrol has been undertaken for decades to target *Ae. aegypti* but it is challenging to apply the copepods as most of the larval habitats are not favourable habitats for these copepods [183, 184]. Other mosquito predators, such as fish, water bugs and frogs, may play significant roles in biocontrol in the future [185].

As biocontrol agents, entomopathogenic fungi, bacteria and viruses have been developed to specifically kill mosquitoes. The most commonly used microorganism is* Bti* which destroys the gut of the mosquito larvae by producing δ-endotoxin [186]. Several studies have indicated the lethal effect of entomopathogenic fungi, such as *Metarhizium anisopliae*, to adult mosquitoes [187]. The fungi sporulate to penetrate the cuticle of the mosquitoes, resulting in the death of mosquitoes by obliteration of tissues as well as the toxins produced [188].

Another method includes the release of genetically modified mosquitoes that have been infected with *Wolbachia* sp., an endosymbiotic bacteria [189] and of sterile-male mosquitoes [190]. The cytoplasmic incompatibility induced by *Wolbachia* sp. causes sterility, thereby suppressing mosquito populations. The presence of *Wolbachia* strain, *w*MelPop, reduces the adult lifespan* via* the inhibition of pathogen replication as well as the upregulation of immune genes [191, 192]. The sterile insect technique (SIT) can also be manipulated* via* genetically engineered sterile male mosquitoes, such as OX513A (which carries a repressible dominant lethal transgene insertion that causes lethality at the late larval or early pupal stages). These released sterile males subsequently mate with wild females, and the resultant offspring will die before adult metamorphosis which reduces their reproductive potential. Ultimately, field trials and further research on the sustainability and cost-effectiveness of both approaches will be necessary.

In summary, the prevalence of dengue fever and increasing trend of resistance towards different categories of insecticides are alarming in many Southeast Asian countries. A well-researched understanding of the mechanism of resistance and susceptibility of the mosquitoes is of utmost importance for the development of an effective control method of *Aedes* mosquitoes in these endemic regions.

## Supplementary Information

**Additional file 1: Table S1.** Summary of characteristics data of the included studies.

## Data Availability

Not applicable.

## Abbreviations

AChE: Acetylcholinesterase
*Bti*: *Bacillus thuringiensis israelensis*
CHC: Cuticular hydrocarbon
CPR: NADPH-cytochrome P450 reductase
CYP: Cytochrome P450
DDT: Dichlorodiphenyltrichlorethane
F: Phenylalanine
GABA: γ-Aminobutyric acid
GST: Glutathione* S*-transferases
H: Histidine
*kdr*: Knockdown resistance
L: Leucine
LLINs: Long-lasting insecticide nets
PBA1c: 3-Phenoxybenzyl alcohol
PBA1d: 3-Phenoxybenzaldehyde
PBAcid: 3-Phenoxybenzoic acid
*RDL*: Resistance to dieldrin gene
S: Serine
Vssc: Voltage-sensitive sodium channel
