# Supplementary material for: Dengue fever and insecticide resistance in Aedes mosquitoes in Southeast Asia: a review
Source: Parasit Vectors. 2021 Jun 10;14:315. doi: 10.1186/s13071-021-04785-4 (PMC8194039; doi:10.1186/s13071-021-04785-4)
Supplement: Supplementary file 1 — Additional file 1: Table S1. Summary of characteristics data of the included studies. [file 13071_2021_4785_MOESM1_ESM.docx]

**Additional file 1: Table S1****. Summary of characteristics data of the included studies**

| **Country** | **Year** | **Insecticide** | **Location** | **Stage** | **References** |
| --- | --- | --- | --- | --- | --- |
| Cambodia | 2001 | Temephos | Phnom Penh  Kampong Cham | Larvae of *Ae. aegypti* | Polson *et al*., 2001 [135] |
|  | 2016 | *Bacillus thuringiensis israelensis* (Bti) (VectoBac WG) | Peani and Ou Ruessei communes, Kampong Tralach district of Kampong Chhnang Province | Pupae and adults of *Ae. aegypti* | Setha *et al*., 2016  [193] |
|  | 2018 | Temephos  Deltamethrin  Permethrin | Urban versus rural:  Phnom Penh  Siem Reap  Kampong Cham  Battambang | Larvae and adults of *Ae. aegypti* | Boyer *et al*., 2018  [133] |
| Indonesia | 2012 | Temephos | Surabaya, Indonesia | Larvae of *Ae. aegypti* | Mulyatno *et al*., 2012 [139] |
|  | 2015 | Deltamethrin Permethrin | Yogyakarta | Adults of *Ae. aegypti* | Wuliandari *et al*., 2015 [140] |
|  | 2016 | α-cypermethrin Deltamethrin λ-cyhalothrin Malathion | Semarang, Java  Kudus, Java  Jepara, Java  Surakarta, Java | Adults of *Ae. aegypti* | Sayono *et al*., 2016 [142] |
|  | 2016 | Temephos Malathion Permethrin | Java, Indonesia  Bogor, Tasikmalaya, Sumedang, Garut  and Semarang | Larvae of *Ae. aegypti* | Putra *et al*., 2016 [146] |
|  | 2017 | Permethrin | Jakarta  (Kebon Jeruk, Kebayoran Lama, Kebayaron Baru, Cempaka Putih, Kramat Jati, Makasar, Ciracas, Cipayung) | Adults of *Ae. aegypti* | Hamid *et al*., 2017 [143] |
|  | 2017 | Malathion Deltamethrin Permethrin  λ-cyhalothrin Bendiocarb Cyflothrin | Denpasar, Bali | Adults of *Ae. aegypti* | Hamid *et al*., 2017 [141] |
|  | 2018 | Temephos | Padang, Sumatra  (Jati, Gunung Pangilun, Lubuk Minturun, Korong Gadang, Bandar Baut) | Larvae of *Ae. aegypti* | Hasmiwati *et al*., 2018 [145] |
|  | 2019 | Bromophos Chlopyrifos Fenitrothion  Fenthion Malathion Temephos DDT  Dieldrin | Kuningan, Java  Padang, Java  Samarinda, Borneo  Pontianak, Borneo  Denpasar, Bali  Mataram, Lombok  Dompu, Sumbawa  Manggarai Barat, Flores  East Sumba, Sumba  South Central Timor, Timor | Larvae of *Ae. aegypti* | Haziqah-Rashid *et al*., 2019 [138] |
|  | 2019 | Pyrethroid | Kuningan, Java  Padang, Java  Samarinda, Borneo  Pontianak, Borneo  Denpasar, Bali  Mataram, Lombok  Dompu, Sumbawa  Manggarai Barat, Flores  East Sumba, Sumba | Adults of *Ae. aegypti* | Amelia-Yap *et al*., 2019 [147] |
|  | 2019 | Pyrethroids (permethrin) | Central Java  Rejowinangun Utara  Gelangan  Cacaban  Magelang | Adults of *Ae. aegypti* | Satoto *et al*., 2019 [194] |
|  | 2020 | Malathion  Deltamethrin  Permethrin  Lambda-cyhalothrin  Bendiocarb  Cyflothrin | Makassar, Sulawesi | Adults of *Ae. aegypti* | Hamid *et al*., 2020 [144] |
| Laos | 2018 | DDT  Malathion  Permethrin  Deltamethrin Temephos | Vientiane Capital Luang Prabang | Larvae and adults of *Ae. albopictus* | Tangena *et al*., 2018 [149] |
|  | 2018 | *Bacillus thuringiensis israelensis* (Bti)  Diflubenzuron Pyriproxyfen Spinosad | Vientiane, Laos | Larvae of *Ae. aegypti* | Marcombe *et al*., 2018 [151] |
|  | 2019 | Malathion  Temephos  DDT  Permethrin  Deltamethrin | Xayaboury  Luang Prabang  Vientiane Capital  Saravane  Attapeu | Larvae and adults of *Ae. aegypti* | Marcombe *et al*., 2019 [150] |
| Malaysia | 2005 | Temephos | Selangor  Taman Samudera, Gombak  Kampung Banjar,  Gombak |  | Chen *et al*., 2005  [197] |
|  | 2005 | Temephos | Selangor  Taman Samudera, Gombak  Kampung Banjar, Gombak  Kuala Lumpur  Taman Lembah Maju, Cheras Kampung Baru, City  centre | Larvae of *Ae. aegypti* and *Ae. albopictus* | Chen *et al*., 2005  [196] |
|  | 2005 | Pyriproxyfen (Sumilarv 0.5G) | Laboratory and simulated field trial | Larvae of *Ae. aegypti* aNd *Ae. albopictus* | Vythilingam *et al*., 2005 [197] |
|  | 2006 | *Bacillus thuringiensis israelensis (Bti)* | Penang Island  Sungai Nibong  Sungai Dua  Air Itam  Bayan Lepas Kampung Serani Ujung Batu | Adults of *Ae. aegypti* and *Ae. albopictus* | Lee *et al*., 2006 [198] |
|  | 2008 | *Bacillus thuringiensis israelensis (Bti), VectoBac WG* | Selangor  Suburban residential area (TST)  Temporary settlement site (KB) | Larvae of *Ae. aegypti* and *Ae. albopictus* | Lee *et al*., 2008  (abstract only) [199] |
|  | 2010 | *Bacillus thuringiensis israelensis* (Bti) Temephos | Selangor  Shah Alam: Sites A and B | Larvae of *Ae. aegypti* | Loke *et al*., 2010 [200] |
|  | 2011 | Permethrin  Deltamethrin | Penang island  Sungai Dua  Persiaran Mayang  Pasir | Adult *Ae. albopictus* | Chan *et al*., 2011 [201] |
|  | 2012 | Permethrin  Cyfluthrin Malathion Fenitrothion Propoxur Bendiocarb  DDT | Shah Alam | Adults of *Ae. aegypti* | Rong *et al*., 2012  (abstract only) [202] |
|  | 2013 | Temephos | Taman Samudera, Gombak  Banjar, Gombak | Larvae of *Ae. albopictus* | Chen *et al*., 2013 [203] |
|  | 2014 | Deltamethrin Permethrin Fenitrothion Malathion | Kampar, Perak  Taman Kampar Jaya  Taman Juloong | Adults of *Ae. albopictus* | Ho *et al*., 2014 [204] |
|  | 2015 | Lambda-cyhalothrin Pirimiphos-methyl | Bagan Dalam, Penang | Adults of *Ae. aegypti* and *Ae. albopictus* | Abu Hasan *et al*., 2015 [155] |
|  | 2015 | Permethrin Deltamethrin  DDT  Dieldrin Bendiocarb  Malathion | Penang  Kuala Lumpur  Johor Bharu  Kota Bharu | Adults of *Ae. aegypti* and *Ae. albopictus* | Ishak *et al*., 2015 [66] |
|  | 2015 | Dieldrin | Selangor  Petaling Jaya | Adults of *Ae. albopictus* | Low *et al*., 2015  (abstract only) [205] |
|  | 2016 | Temephos  Malathion | Penang  Sungai Nibong  Gelugor  Balik Pulau  Permatang Damar Laut  Pintasan Bahagia | Larvae of *Ae. albopictus* | Rahim *et al*., 2016 [206] |
|  | 2016 | Dieldrin  Malathion  Bendiocarb  DDT  Deltamethrin  Permethrin | Penang  Kuala Lumpur  Johor Bharu  Kota Bharu | Adults of *Ae. albopictus* | Ishak *et al*., 2016 [107] |
|  | 2017 | Malathion  Permethrin  Deltamethrin  DDT | Penang  Sungai Nibong  Gelugor  Balik Pulau  Permatang Damar Laut  Pintasan Bahagia | Larvae of *Ae. albopictus* | Rahim *et al*., 2017 [207] |
|  | 2017 | Permethrin  Deltamethrin  DDT  Bendiocarb  Malathion | Penang  Kuala Lumpur  Kota Bharu  Johor Bharu | Adults of *Ae. aegypti* | Ishak *et al*., 2017 [157] |
|  | 2017 | Permethrin | Selangor  Melaka  Kedah  Johor  Perlis | Adults of *Ae. aegypti* | Rasli et al., 2017 [152] |
|  | 2018 | Deltamethrin Permethrin  Cyfluthrin  Lambda-cyhalothrin  Malathion Fenitrothion | Taman Seri Bayu Flat Camar  Taman Dahlia  Ridzuan Condominium | Adults of *Ae. aegypti* | Rasli *et al*., 2018 [153] |
|  | 2018 | DDT  Propoxur Malathion  Temephos Cyfluthrin Deltamethrin  Etofenprox Lambdacyhalothrin  Permethrin | Selangor  Hulu Selangor  Gombak  Hulu Langat  Kuala Langat  Kuala Selangor  Petaling  Klang  Sabak Bernam  Sepang | Larvae of *Ae. aegypti* | Leong *et al*., 2018 [154] |
|  | 2018 | Bromophos  Malathion  Fenthion  Fenitrothion,  Temephos  Chlorpyrifos  DDT  Dieldrin | Sabah  Kudat  Kota Marudu  Kota Belud  Tuaran  Kota Kinabalu  Penampang  Putatan  Papar | Larvae of *Ae. aegypti* | Elia-Amira *et al*., 2018 [158] |
|  | 2019 | DDT  Cyfluthrin  Deltamethrin  Etofenprox  Lambdacyhalothrin  Permethrin  Malathion  Propoxur | Selangor  Hulu Selangor  Gombak  Hulu Langat  Kuala Langat  Kuala Selangor  Petaling  Klang  Sabak Bernam  Sepang | Adults of *Ae. aegypti* | Leong *et al*., 2019 [156] |
|  | 2019 | Cyfluthrin  Lambda-cyaholthrin  Deltamethrin  Dieldrin  Malathion  Bendiocarb  Propoxur  DDT  Fenitrothion | Sabah  Kudat  Kota Marudu  Kota Belud  Tuaran  Kota Kinabalu  Penampang  Putatan  Papar  Keningu  Tenom  Tawau  Kunak  Sandakan  Beluran | Adults of *Ae. albopictus* | Elia-Amira *et al*., 2019 [159] |
| Myanmar | 2014 | d-allethrin | Yangoon city | Larvae of *Ae. aegypti* | Kawada *et al*., 2014 [160] |
|  | 2018 | Pyriproxyfen resin discs (SumiLarv®2MR) | Hlaing Thar Yar Township, Yangon | Larvae of *Ae. aegypti* | Oo *et al*., 2018 [208] |
| Philippines | 2020 | Pyrethroids | Bangkok, Thailand  Cebu, Philippines | Adults of *Ae. aegypti* | Cosme *et al*., 2020 [209] |
| Singapore | 2001 | *Bacillus thuringiensis israelensis*  (Vectobacâ 12 AS) Pirimiphos-methyl  (Actellicâ 50 EC) | Island of Singapore | Larvae and adults of *Ae. aegypti* | Chung *et al*., 2001 [210] |
|  | 2014 | Permethrin  Temephos  Pirimiphos methyl | Pyrethroid-resistant population, SPS0, collected from  Singapore in 2009 | Adults and larvae of *Ae. aegypti* | Kasai *et al*., 2014 [128] |
|  | 2014 | Permethrin Deltamethrin | Pyrethroid-resistant population, SP, collected from  Singapore in 2009 | Adults of *Ae. aegypti* | Hirata *et al*., 2014 [166] |
|  | 2015 | Deltamethrin | Five regions of  Singapore | Adults of *Ae. aegypti* | Pang *et al*., 2015 [71] |
|  | 2016 | Pyrethroids | Serogoon, Singapore | Adults of *Ae. albopictus* | Xu *et al*., 2016 [76] |
| Thailand | 2009 | Temephos  Cypermethrin  Alpha-cypermethrin  Zeta-cypermethrin  Deltamethrin  Etofenprox  Zetamethrin + Dichlorvos | North-east Thailand  Amnat Charoen, Kalasin, Mukdahan,  Nakhon Phanom, Sakon Nakhon, Si Sa Ket, Ubon Ratchathani and Yasothon | Larvae and adults of *Ae. aegypti* | Pimsamarn *et al*., 2009 [174] |
|  | 2011 | Permethrin Deltamethrin Lambda-cyhalothrin | Bangkok  Kanchanaburi  Chonburi  Chanthaburi  Prachinburi  Nakhon Ratchasima Buri Ram  Surin  Si Sa Ket  Kalasin  Roi Et  Udon Thani  Khon Kaen  Phang Nga  Phuket  Phatthalung  Surat Thani  Chumphon  Prachuap Khiri Khan  Songkhla  Tak  Nakhon Sawan  Uthai Thani  Phrae  Chiang Rai  Lampang  Kamphaeng Phet  Chiang Mai  Lamphun | Adults of *Ae. aegypti* and *Ae. albopictus* | Chuaycharoensuk *et al*., 2011 [211] |
|  | 2011 | Permethrin  Deltamethrin Cyfluthrin  Malathion  DDT | 36 districts of Metropolitan Bangkok | Adults of *Ae. aegypti* | Komalamisra *et al*., 2011 [212] |
|  | 2013 | Alpha-cypermethrin  Lambda-cyhalothrin Deltamethrin | Pu Teuy Village, Kanchanaburi Province | Adults of *Ae. aegypti* | Manda *et al*., 2013 [213] |
|  | 2013 | Deltamethrin | Chiang Rai  Mae Jai  Lampang  Phrae  Khon Kaen  Sra Gaew  Chanthaburi  Trat  Nakhon Si Thammarat  Rayong  Mae Sot  Mae Sariang  Lamphun  Chiang Mai  Mae Sun | Adults of *Ae. aegypti* | Stenhouse *et al*., 2013 [67] |
|  | 2014 | Temephos | North Thailand  Chiang Mai  Central Thailand  Nakhon Sawan  South Thailand  Phatthalung | Larvae of *Ae. aegypti* | Poupardin *et al*., 2014 [99] |
|  | 2015 | Deltamethrin  Permethrin  Bifenthrin  cCpermethrin  alpha-cypermethrin | Rayong  Koh Chang, Trat  Pong Nom Ron, Chanthaburi | Adults of *Ae. albopictus* | Thanispong *et al*., 2015 [175] |
|  | 2016 | Deltamethrin | Chiang Mai city | Adults of *Ae. aegypti* | Plernsub *et al*., 2016 [171] |
|  | 2019 | Transfluthrin | Central Thailand  Muang, Nonthaburi | Adults of *Ae. aegypti* | Sukkanon *et al*., 2019 [214] |
|  | 2019 | Deltamethrin  Deltamethrin + piperonyl butoxide | North Thailand  Lampang  Phrae  West Thailand  Phetchaburi  East Thailand  Rayong  Northeast Thailand  Loei  Udon Thani  Nong Bua Lam Phu South Thailand  Phatthalung | Adults of *Ae. aegypti* | Kongmee *et al*., 2019 [215] |
|  | 2020 | Deltamethrin  Cypermethrin | Eastern Thailand  Rayong  Chanthaburi | Adults of *Ae. aegypti* | Sathantriphop *et al*., 2020 [216] |
|  | 2020 | Temephos | 62 locations  Central Thailand  Bangkok  Kanchanaburi  Nakhon Pathom  Suphan Buri  Phetchaburi  Prachuap Khiri Khan  Ang Thong  Chanthaburi  Chon Buri  Lop Buri  Samut Prakan  Sing Buri  Trat  North Thailand  Lampang  Nan  Phrae  Nakhon Sawan  Phichit  Uthai Thani  Phetchabun  Phitsanulok  Northeast Thailand  Roi Et  Ubon Ratchathani  Loei  Nong Bua Lam Phu  Udon Thani  Kalasin  Maha Sarakham  South Thailand  Phatthalung  Satun  Trang | Larvae of *Ae. aegypti* | Saeung *et al*., 2020 [217] |
|  | 2020 | Pyrethroids | Bangkok, Thailand  Cebu, Philippines | Adults of *Ae. aegypti* | Cosme *et al*., 2020 [209] |
| Vietnam | 2009 | Allethrin | Mekong Delta | Larvae of *Ae. aegypti* | Kawada *et al*., 2009 [180] |
|  | 2018 | Permethrin Deltamethrin  Lambda-cyhalothrin DDT  Propoxur  Malathion | Ha Noi  Thanh Hoa  Nghe An  Ha Tinh  Khanh Hoa | Adults of *Ae. aegypti* | Lien *et al*., 2018 [218] |
|  | 2019 | DDT  Propoxur  Malathion  Deltamethrin  Lambda-cyhalothrin Permethrin | Khanh Hoa Province | Adults of *Ae. aegypti* | Lien *et al*., 2019 [219] |
|  | 2019 | Permethrin  Etofenprox  Deltamethrin | Tu Liem, Hanoi  Bat Trang, Hanoi  Ba Vi, Hanoi  Ho Chi Minh city  Cat Tien National Park, Dong Nai  Yok Don National Park, Dak Lak  Buon Ma Thuot, Dak Lak  Hoa Kien, Phu Yen | Adults of *Ae. albopictus* | Kasai *et al*., 2019 [167] |

**References**

1. Setha T, Chantha N, Benjamin S, Socheat D. Bacterial larvicide, *Bacillus thuringiensis israelensis* strain AM 65–52 water dispersible granule formulation impacts both dengue vector, *Aedes aegypti* (L.) population density and disease transmission in Cambodia. PLoS Negl Trop Dis. 2016;10(9):e0004973. https:// doi. org/ 10. 1371/ journ al. pntd. 00049 73.
2. Satoto TBT, Satrisno H, Lazuardi L, Diptyanusa A, Purwaningsih, Rumbiwati, et al. Insecticide resistance in *Aedes aegypti*: an impact from human urbanisation? PLoS ONE. 2019;14(6):e0218079. https:// doi. org/10. 1371/ journ al. pone. 02180 79.
3. Chen CD, Nazni WA, Lee HL, Sofian-Azirun M. Weekly variation on susceptibility status of *Aedes* mosquitoes against temephos in Selangor, Malaysia. Trop Biomed. 2005;22(2):195–206.
4. Chen CD, Nazni WA, Lee HL, Sofian-Azirun M. Susceptibility of *Aedes aegypti* and *Aedes albopictus* to temephos in four study sites in Kuala Lumpur City Centre and Selangor State, Malaysia. Trop Biomed. 2005;22:207–16.
5. Vythilingam I, Luz BM, Hanni R, Beng TS, Huat TC. Laboratory and field evaluation of the insect growth regulator pyriproxyfen (Sumilarv 0.5G) against dengue vectors. J Am Mosq Control Assoc. 2005;21(3):296–300. https:// doi. org/ 10. 2987/ 8756- 971X(2005) 21[296: LAFEOT] 2.0. CO;2.
6. Lee YW, Zairi J. Susceptibility of laboratory and field-collected *Aedes aegypti* and *Aedes albopictus* to *Bacillus thuringiensis israelensis* H-14. J Am Mosq Control Assoc. 2006;22(1):97–101. https:// doi. org/ 10. 2987/8756- 971X(2006) 22[97: SOLAFA] 2.0. CO;2.
7. Lee HL, Chen CD, Masri SM, Chiang YF, Chooi KH, Benjamin S. Impact of larviciding with a *Bacillus thuringiensis israelensis* formulation, VectoBac WG, on dengue mosquito vectors in a dengue endemic site in Selangor State, Malaysia. Southeast Asian J Trop Med Public Health.

2008;39(4):601–9.

1. Loke SR, Andy-Tan WA, Benjamin S, Lee HL, Sofian-Azirun M. Susceptibility of field-collected *Aedes aegypti* (L.) (Diptera: Culicidae) to *Bacillus thuringiensis israelensis* and temephos. Trop Biomed. 2010;27(3):493–503.
2. Chan HH, Mustafa FF, Zairi J. Assessing the susceptibility status of *Aedes albopictus* on Penang Island using two different assays. Trop Biomed. 2011;28(2):464–70.
3. Rong LS, Ann AT, Ahmad NW, Lim LH, Azirun MS. Insecticide susceptibility status of field-collected *Aedes* (Stegomyia) *aegypti* (L.) at a dengue endemic site in Shah Alam, Selangor, Malaysia. Southeast Asian J Trop Med Public Health. 2012;43(1):34–47.
4. Chen CD, Nazni WA, Lee HL, Norma-Rashid Y, Lardizabal ML, Sofian-Azirun M. Temephos resistance in field *Aedes* (Stegomyia) *albopictus* (Skuse) from Selangor, Malaysia. Trop Biomed. 2013;30(2):220–30.
5. Ho LY, Loh TS, Yam LA. Surveillance and resistance status of *Aedes* population in two suburban residential areas in Kampar town, Perak, Malaysia. Trop Biomed. 2014;31(3):441–8.
6. Low VL, Vinnie-Siow WY, Lim YAL, Tan TK, Leong CS, Chen CD, et al. First molecular genotyping of A302S mutation in the gamma aminobutyric acid (GABA) receptor in *Aedes albopictus* from Malaysia. Trop Biomed. 2015;32(3):554–6.
7. Rahim J, Ahmad AH, Kassim NF, Ahmad H, Ishak IH, Rus AC, et al. Revised discriminating lethal doses for resistance monitoring program on *Aedes albopictus* against temephos and malathion in Penang Island, Malaysia. J Am Mosq Control Assoc. 2016;32(3):210–6. https:// doi. org/10. 2987/ 16- 6556.1.
8. Rahim J, Ahmad AH, Ahmad H, Ishak IH, Rus AC, Maimusa HA. Adulticidal susceptibility evaluation of *Aedes albopictus* using new diagnostic doses in Penang Island, Malaysia. J Am Mosq Control Assoc. 2017;33(3):200–8. https:// doi. org/ 10. 2987/ 16- 6607R.1.
9. Oo SZM, Thaung S, Maung YNM, Aye KM, Aung ZZ, Thu HM, et al. Effectiveness of a novel long-lasting pyriproxyfen larvicide (SumiLarv2MR) against *Aedes* mosquitoes in schools in Yangon, Myanmar. Parasites Vectors. 2018;11(1):16. https:// doi. org/ 10. 1186/ s13071- 017- 2603-9.
10. Cosme LV, Gloria-Soria A, Caccone A, Powell JR, Martins AJ. Evolution of kdr haplotypes in worldwide populations of *Aedes aegypti*: independent origins of the F1534C kdr mutation. PLoS Negl Trop Dis. 2020;14(4):e0008219. https:// doi. org/ 10. 1371/ journ al. pntd. 00082 19.
11. Chung YK, Lam-Phua SG, Chua YT, Yatiman R. Evaluation of biological and chemical insecticide mixture against Aedes aegypti larvae and adults by thermal fogging in Singapore. Med Vet Entomol. 2001;15(3):321–7. https:// doi. org/ 10. 1046/j. 0269- 283x. 2001. 00311.x.
12. Chuaycharoensuk T, Juntarajumnong W, Boonyuan W, Bangs MJ, Akratanakul P, Thammapalo S, et al. Frequency of pyrethroid resistance in *Aedes aegypti* and *Aedes albopictus* (Diptera: Culicidae) in Thailand. J Vector Ecol. 2011;36(1):204–12. https:// doi. org/ 10. 1111/j. 1948- 7134.2011. 00158.x.
13. Komalamisra N, Srisawat R, Phanbhuwong T, Oatwaree S. Insecticide susceptibility of the dengue vector, *Aedes aegypti* (L.) in Metropolitan Bangkok. Southeast Asian J Trop Med Public Health. 2011;42(4):814–23.
14. Manda H, Shah P, Polsomboon S, Chareonviriyaphap T, Castro-Llanos F, Morrison A, et al. Contact irritant responses of *Aedes aegypti* using sublethal concentration and focal application of pyrethroid chemicals. PLoS Negl Trop Dis. 2013;7(2):e2074. https:// doi. org/ 10. 1371/ journ al.pntd. 00020 74.
15. Sukkanon C, Bangs MJ, Nararak J, Hii J, Chareonviriyaphap T. Discriminating lethal concentrations for transfluthrin, a volatile pyrethroid compound for mosquito control in Thailand. J Am Mosq Control Assoc. 2019;35(4):258–66. https:// doi. org/ 10. 2987/ 19- 6832.1.
16. Kongmee M, Thanispong K, Sathantriphop S, Sukkanon C, Bangs MJ, Chareonviriyaphap T. Enhanced mortality in deltamethrin-resistant *Aedes aegypti* in Thailand using a piperonyl butoxide synergist. Acta Trop. 2019;189:76–83. https:// doi. org/ 10. 1016/j. actat ropica. 2018. 09. 025.
17. Sathantriphop S, Paeporn P, Ya-Umphan P, Mukkhun P, Thanispong K, Chansang C, et al. Behavioral action of deltamethrin and cypermethrin in pyrethroid-resistant *Aedes aegypti* (Diptera: Culicidae): implications for control strategies in Thailand. J Med Entomol. 2020;57(4):1157–67. https:// doi. org/ 10. 1093/ jme/ tjaa0 19.
18. Saeung M, Ngoen-Klan R, Thanispong K, Muenworn V, Bangs MJ, Chareonviriyaphap T. Susceptibility of *Aedes aegypti* and *Aedes albopictus* (Diptera: Culicidae) to temephos in Thailand and surrounding countries. J Med Entomol. 2020;57(4):1207–20. https:// doi. org/ 10. 1093/ jme/ tjaa035.
19. Lien NTK, Ngoc NTH, Hien NT, Hoang NH, Binh NTH. Two novel mutations in the voltage-gated sodium channel associated with knockdown resistance (kdr) in the dengue vector *Aedes aegypti* in Vietnam. J Vector Ecol. 2018;43(1):184–9. https:// doi. org/ 10. 1111/ jvec. 12298.
20. Lien NTK, Ngoc NTH, Lan NN, Hien NT, Tung NV, Ngan NTT, et al. Transcriptome sequencing and analysis of changes associated with insecticide resistance in the dengue mosquito (*Aedes aegypti*) in Vietnam. Am J Trop Med Hyg. 2019;100(5):1240–8. https:// doi. org/ 10. 4269/ ajtmh.18- 0607.
